# Can pre-collected register data be used to identify dairy herds with good cattle welfare?

**DOI:** 10.1186/1751-0147-53-S1-S8

**Published:** 2011-06-20

**Authors:** Ann-Kristin Nyman, Ann Lindberg, Charlotte Hallén Sandgren

**Affiliations:** 1 National Veterinary Institute, SE-751 89 Uppsala, Sweden; 2 Swedish Dairy Association, Box 210, SE- 101 24 Stockholm, Sweden

## Abstract

**Abstract:**

**Background**

Pre-recorded register data from dairy herds are available in almost all Nordic countries. These databases can be used for research purposes, and one of the research areas is animal welfare. The aim of this study was to investigate if pre-recorded register data could be used to identify herds with good welfare, and to investigate if a combination of register data sets could be used to be able to more correctly distinguish between herds with good welfare and herds with welfare deficiencies.

**Methods**

As a first step, nine animal-based measurements on calves, young stock and cows in 55 randomly selected herds were performed on-farm as the basis for a classification of welfare at the herd level. The definition for being a case herd with “good welfare” was no score lying among the 10% worst in any of the nine welfare measurements. Twenty-eight of the 55 herds were cases according to this definition. As a second step, 65 potential welfare indicators, based on register data in a national dairy database, were identified by expert opinion. In the final step, the extent to which the suggested welfare indicators predicted farms’ as having good welfare according to the stated definition was assessed. Moreover, the effect of combining in sequence a previously developed model that identified herds with poor welfare with the present model identifying herds with good welfare was investigated.

**Results**

The final set of welfare indicators used to identify herds with good animal welfare included two fertility measures, cow mortality, stillbirth rate, mastitis incidence and incidence of feed-related diseases (including gastrointestinal disturbances but excluding paralyses and cramps). This set had a test sensitivity of correctly classifying herds with no score lying among the 10% worst of the nine welfare measurements of 96 %. However, the specificity of the test was only 56% indicating difficulties for the test to correctly classifying herds with one or more scores lying among the 10% worst. Combining the previously developed model with the present model, improved the welfare classification.

**Conclusions**

This study shows that pre-collected register data may be used to give approval to dairy farms with “good welfare” and that combining different sets of register data can improve the classification of herd welfare.

## Background

All Nordic countries except Iceland have national dairy disease recoding systems that rely on the reporting of veterinary-treated disease events [1-3]. In Sweden, only veterinarians are allowed to start an antimicrobial treatment and it is compulsory for veterinarians to report these treatments to the Swedish animal disease recording scheme (SADRS), which is administered by the Swedish Board of Agriculture. The animal disease recording system is linked to the Swedish official milk recording scheme (SOMRS) in which herds are enrolled on a voluntary basis. The data in these databases are primarily meant to be used by the farmers and veterinarians, and by the Swedish Board of Agriculture, but the amount of data available in the databases also makes them attractive for research purposes. The Swedish Dairy Association has developed different tools that use the information in these databases in advisory services, such as in breeding programs, feeding schemes and health plans [4].

Today there is considerable agreement regarding the use of animal-based measures such as lameness, body condition and cleanliness in assessments of animal welfare in dairy herds [5,6]. However, these measures are time- and labour demanding and there are concerns whether a welfare assessment system based on such measures can feasibly be implemented in practice if a large number of herds are to be monitored on a regular basis. If such a system was needed, a more efficient way of assessing the animal welfare would be necessary, and using the available data in the SADSR and SOMRS databases is one possible solution.

The aim of this study was to investigate if pre-recorded register data could be used to identify herds with good welfare, and to investigate if a combination of register data sets could be used to distinguish more correctly between herds with good welfare and herds with welfare deficiencies.

## Methods

### Study herds

Herds eligible for inclusion in the study were Swedish dairy herds enrolled in the Swedish official milk recording and animal disease recording schemes (SOMRS and SADRS) in 2004. The study population was selected to reflect expectations regarding the herd size in 2010. Based on data from 1995 – 2004 an expected median herd size was estimated for 2010. To establish a similar distribution for herd size for the estimation of 2010 as of 1995 – 2004 stratification was used. The distribution of 1995 – 2004 was stratified in 10 strata making the proportion of herds in each stratum equal. These strata and proportions were then used to establish the expected distribution in 2010. The estimated median herd size and herd size in the 10 strata of 2010 were 65 cows, and 15-23, 24-30, 31-35, 36-40, 41-46, 47-52, 53-60, 61-72, 73-93, >93, respectively. The participating herds were then randomly selected from all dairy herds delivering milk in two selected areas, one in southern and one in northern Sweden, ensuring equal proportion in the set strata. The randomly selected herds were contacted and ask if they were willing to participate. Out of 64 contacted herds 62 accepted the invitation to participate and were visited twice during 2005, the first visit occurring in March to mid-June and the second in October to December. Of the 62 herds 55 had complete records in the SOMRS and in the SADRS, and only these were included in the final statistical analyses.

### Field assessment of animal welfare

The methodology used to assess animal welfare within the study herds has previously been described [7], but briefly nine animal-based welfare measurements were assessed on farm during two farm visits; cleanliness and body condition in calves, cows and young stock, in combination with lameness, injuries/inflammations, and rising behaviour which were recorded only for cows. At each visit two assessors, independently and without communicating, performed the assessment. At the second visit one of the previous assessors was replaced, so that each farm was visited by three assessors, one of whom visited the farm twice. A total of 8 assessors participated in the study, all assessing all parameters.

### The gold standard for good animal welfare

Herd-level estimates of the animal-based measurements were obtained by applying cut-off levels and calculating the proportion of animals within each age group that exceeded the cut-off. The welfare “gold standard” i.e. the definition of welfare status (no welfare remarks vs. one or more welfare remarks) against which the performance of potential welfare indicators in the database was to be evaluated, was based on the number of animal-based measurements where a herd did not score among the 10% worst. A score among the 10% worst gave the herd a ‘remark’ for that measure, and a herd with zero scores among the 10% worst was defined as being a herd with good welfare.

### Register data

The database of the Swedish national dairy recording system (hereafter called the Swedish Cattle Database (SCD) in which data from the SOMRS and SADRS are merged was used as the main source of the potential welfare indicators for this study. The SCD includes information on e.g. fertility, genetics, diseases, mortality including culling reasons, production and slaughterhouse registrations as well as demographic data [2,8]. The coverage of the database in 2005 included 78% of the dairy herds in Sweden, representing 83% of the Swedish dairy cows [9,10]. Additional data on cattle mortality (enabling identification of euthanized and fallen stock) was retrieved from the Board of Agriculture, where a registry of all Swedish cattle is kept, in accordance with EU Directive 1760/2000. This was done in order to investigate the usefulness of such data in case a herd is not affiliated to the SOMRS and because the SCD (at the time) did not have “euthanasia” and “fallen stock” as separate culling codes.

#### Choice of potential welfare indicators from the register data

The potential welfare indicators were calculated for the period from January to December 2005, i.e. over the same period of time as the on-farm data were collected, for the 55 farms with complete records. The selection of potential welfare indicators has also been described earlier [7], but will be described briefly. Seven different focus areas reflecting important components of animal welfare and covering the complete life-span of a dairy cow were suggested by a group of experts on quality programmes, marketing, dairy farm economy and animal health within the Swedish Dairy Association. The seven areas were; management, calves and young stock, survival/intensity of production, feeding, udder health, claw and leg health, and drug use. In a second step, a total of 65 potential welfare indicators from the SCD and the Board of Agriculture register data that could be expected, in various degrees, to reflect animal welfare aspects of the focus areas were identified by a group consisting of twelve national experts in animal health, welfare, production and epidemiology.

### Data analyses

#### Variable reduction

The goal of the analyses was not to study causality, nor to determine exact relationships between animal-based measurements and potential welfare indicators. Rather, the aim was to identify a limited set of pre-recorded welfare indicators that could, in combination, be used to identify herds with possible good animal welfare. Consequently, it was necessary to reduce the initial set of potential welfare indicators suggested by the experts. Therefore, as a first step, univariable associations between all 65 potential welfare indicators and each of nine animal-based measurements were screened using linear regression. Welfare indicators with an association significant at P < 0.05 were then taken forward to a multivariable reduction step, using the same methodology. By including the second step we added a stronger requirement that the potential indicator should show a significant multivariable association with one or more of the animal based measurements, in order to be taken further. Consequently, only indicators that were significantly (P < 0.05) associated with one or more animal-based measures in this multivariable context were considered to be candidates for the final set of welfare indicators. All statistical analyses were performed using the software Stata® version 10 (Stata Corp., College Station, TX, US).

#### Selection of final set of welfare indicators (the “register test tool”)

Each potential welfare indicator was treated as a “diagnostic test”, i.e. a tool that distinguishes between two different statuses; e.g. sick vs. healthy; good vs. poor welfare etc. Like in any diagnostic test measured on a continuous scale, cut-off levels needed to be identified as these are the points at which the “test” would be regarded as “positive”. Because the choice of cut-off level affects “test” performance, measured as sensitivity (the probability of correctly identifying case herds) and specificity (the probability of correctly identifying herds that are not cases), we evaluated each potential welfare indicator at three different levels. The cut-off levels evaluated were the 20^th^, 10^th^ and 5^th^ percentile for welfare indicators that were associated with the animal-based measurements. In this way, all potential welfare indicators were dichotomised (positive or not) into three different “tests” and the sensitivity and specificity for each “test” in identifying a herd “positive” for good welfare were then estimated. By this non-statistical, but systematic, selection procedure, the set of potential welfare indicators was then applied to the study herds, and a herd was regarded as being a case (herd with good welfare) if it was positive on this test. The overall performance of the identified set of welfare indicators was evaluated in terms of sensitivity, specificity and percentage of all herds classified correctly with respect to the field assessed welfare status (good vs. not good). Finally, in an attempt to increase the specificity of the previously published test tool for identification of herds with poor welfare [7], this was combined with the currently identified test set to identify herds with good welfare, increasing the possibility to more correctly distinguish between herds with truly, according to the field assessment, good or poor welfare.

## Results

### Descriptive data

Eighteen of the 55 participating herds were housed in loose housing with cubicle stalls and 37 in tie stalls; six had Swedish Red and White (SRB) cattle, 14 had Swedish Holstein (SHF) breed and 35 had mixed breeds (SRB and SHF). The arithmetic mean annual herd size was 70 cows (ranging from 15 – 415 cows; median = 46 cows).

### Welfare remarks from the field assessment

**Figure 1:**
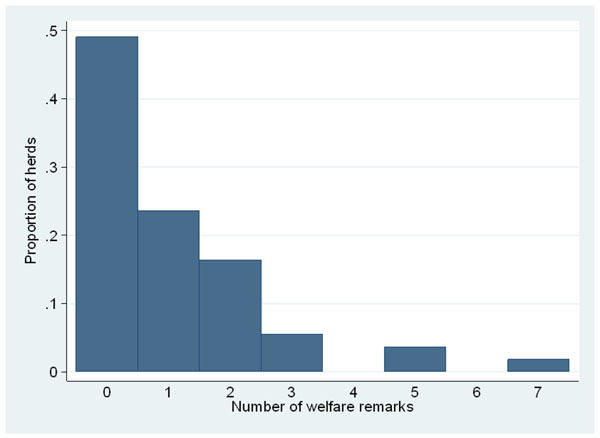
The distribution of number of welfare remarks^1^on nine animal-based measurements used to form a gold standard for defining good welfare in a study involving 55 Swedish dairy herds in 2005. ^2^The animal-based measurements were: cleanliness and body condition in calves, cows and young stock, as well as lameness, injuries/inflammations and rising behaviour (in cows only). ^1^A welfare remark was assigned to the herd if any of the animal-based measurements was above the 90^th^ percentile = 10% worst. ^2^A herd with no measurements above the 90^th^ percentile was considered to be a herd with good welfare (first bar).

Descriptive statistics for the animal-based measurements have previously been described [7]. The range between the 90^th^ and the 100^th^ percentile constituted (10% worst), on average, 38% (23 - 55%) of the total range in herd prevalence of the nine animal-based measurements. Of the 55 herds in the study population, 28 met the criteria for being classified as a herd with good welfare (no remarks). The distribution of number of welfare remarks is shown in figure 1; thirteen herds had two or more remarks, 14 had one remark and 28 had no remark.

### Multivariable analyses

Twenty-eight of the initial 65 potential welfare indicators showed a significant univariable association (*P* < 0.05) with one or more of the nine animal-based welfare measurements. They represented all focus areas except drug use. Following variable reduction using multivariable regression, another 10 indicators were excluded leaving 18 potential welfare indicators for the systematic selection procedure [7].

### Performance of welfare indicators used to identify herds having good welfare

After the systematic selection procedure, six welfare indicators that were jointly able to identify 27 of the 28 case herds (with good welfare according to gold standard definition) were identified. These were; percentage cows with late ongoing artificial inseminations (>120 days) (with a cut-off at the 20^th^ percentile), percentage heifers without mating/artificial insemination by 17 months of age (cut-off: 10^th^ percentile), stillbirth rate (cut-off: 5^th^ percentile), cow mortality (cut-off: 10^th^ percentile), mastitis incidence (cut-off: 10^th^ percentile), and incidence of feed-related diseases (including gastrointestinal disturbances but excluding paralyses and cramps; cut-off: 5^th^ percentile). Herds considered to have good welfare had to have values below the cut-offs in at least one of the welfare indicators. Table 1 gives the estimates of the overall performance of the set into terms of percentage herds correctly classified, sensitivity and specificity. The proportion of test positive herds and predictive value of a positive test are also given. Twelve herds with one or more welfare remarks among the 10% worst were erroneously classified as herds with ”good welfare” by the set of welfare indicators, 6 of these herds had one welfare remark and 6 had two or more welfare remarks.

**Table 1:** Test performance of a set of welfare indicators, used as test tool to identify herds with good welfare. Cut-offs were applied to the distributions of the welfare indicators to produce a 0/1 test result, and these were combined in different sets that were identified through a systematic selection procedure. The parameter sets were applied to 55 Swedish dairy herds involved in a study on dairy cow welfare in 2005. The gold standard consisted of 9 animal-based measurements, where a herd with no welfare remarks above the 90^th^ percentile was regarded as having good welfare.

Performance parameter	Test tool to identify herds with good welfare^1^
Correctly classified (%)	76
Sensitivity	0.96
Specificity	0.56
Test positive (%)	71
Predictive value positive	0.69
Likelihood ratio positive	2.18

^1^ Test set includes following welfare indicators from the register data: Cows with late ongoing AIs, >120 days (cut-off 20^th^ percentile); Heifers not bred >17 months (cut-off: 10^th^ percentile); Stillbirth rate (cut-off: 10^th^ percentile); Cow mortality (cut-off: 10^th^ percentile); Mastitis incidence (cut-off: 10^th^ percentile); Incidence of feed-related diseases (cut-off: 5^th^ percentile)

### Combining the models to identify herds with good or poor welfare

The combination of the model to identify herds with poor welfare with the model to identify herds with good welfare in sequent resulted in herds being classified in to three groups (Table 2); good welfare (no welfare remarks among the 10% worst), uncertain welfare (one welfare remark among the 10% worst), and poor welfare (with ≥ 2 welfare remarks among the 10% worst). The first model classifies herds as either having poor welfare (≥ 2 welfare remarks; 15 herds) vs. not poor welfare (< 2 welfare remarks; 40 herds), and applying the second model only on the 40 herds classified as having < 2 welfare remarks resulted in 32 herds being classified as herds with no welfare remarks and 8 with ≥ 1 welfare remark.

**Table 2:** Distribution of herds classified into different welfare categories by two models using welfare indicators associated with animal-based measurements. The parameter sets were applied to 55 Swedish dairy herds involved in a study on dairy cow welfare in 2005. The gold standard consisted of 9 animal-based measurements, where herds with no welfare remarks above the 90^th^ percentile were regarded as having good welfare, and herds with ≥ 2 welfare remarks above the 90^th^ percentile were regarded as having poor welfare.

(L)Gold standdard classification of herd welfare^1^	Number of herds classified by modelling the welfare indicators in the register data			
		1. Model to classify herds with poor welfare^2^	2. Model to classify herds with good welfare	

	Poor ≥ 2 remark	Not poor < 2 remarks	Not good ≥ 1 remark	Good 0 remarks
1: Good (0 remarks)	1	27	1	26
2: Uncertain (1 remark)	4	10	5	5
3: Poor (≥ 2 remarks)	10	3	2	1

^1^ Number of welfare remarks above the 90^th^ percentile

^2^Model applied to all 55 herds

^3^Model applied to the 40 herds classified as having < 2 remarks above the 90^th^ percentile in the first model

## Discussion

Theoretically this study together with our previous work show that register data can be used to identify herds according to their welfare status. This is very valuable since usage of register data is quick and requires less financial resources than on-farm welfare assessments. However, the methods to identify herds with poor or good welfare have to be validated in field studies to obtain a true measure on how well they actually distinguish between herds with different levels of welfare. For instance, there is a potential risk that smaller dairy herds more often will be identified as herds with poor welfare since the impact of one animal with a deficiency is proportional higher in a small herd than in a larger herd, and this has to be addressed if a national screening is performed. Another concern is the quality of the data in the SADRS and the SOMRS. Studies have shown discrepancies between farmer recordings of disease treatments done by the veterinarians and the veterinary treatments actually registered in the databases [11,12]. Thus, the risk of the farmer choosing a veterinarian not reporting the disease treatments has to be considered if the register tool will be used in schemes aimed at alleviations or rewards directed to herds with presumed good welfare. Additionally, since herds are enrolled in the SOMRS on a voluntary basis there is a risk that farmers displeased with the use of the data in that system will no longer participate.

To identify herds with poor welfare and then herds with good welfare in sequence improved the welfare classification but still resulted in some misclassifications. If only the model to identify herds with < 2 or ≥ 2 welfare remarks were used we could only harshly classify the herds in to two groups, those with presumed poor welfare and those with presumed not poor welfare. Conversely, using only the model which identified herds either with no welfare remarks or with one or more welfare remarks would give an equally harsh classification. Combining the two models resulted in a more refined classification of the herds. Extrapolated to a national level a national screening of herds with these methods would result in approximately 1,500 dairy herds being classified as having poor welfare in Sweden. However, 3,200 herds would be classified as having good welfare, and could get approval for this.

## Conclusions

Register data might be used to identify dairy herds welfare status. However, the developed tools to identify herds with good or poor welfare have to be validated in a field study before nationwide use.

## Competing interests

The authors declare that they have no competing interests.
